# Effects of Sharing Old Pictures With Grandchildren on Intergenerational Relationships: Protocol for a Randomized Controlled Trial

**DOI:** 10.2196/16315

**Published:** 2020-04-30

**Authors:** Zoljargalan Gantumur, Marcos Baez, Nomin-Erdene Ulamnemekh, Francisco Ibarra, Sugarmaa Myagmarjav, Fabio Casati

**Affiliations:** 1 School of Public Health Mongolian National University of Medical Sciences Ulaanbaatar Mongolia; 2 University of Trento Povo (TN) Italy; 3 Université Claude Bernard Lyon 1 Lyon France; 4 School of Engineering Entrepreneurship Tomsk Polytechnic University Tomsk Russian Federation

**Keywords:** intergenerational relationship, social media, Facebook intervention, closeness

## Abstract

**Background:**

Intergenerational relationships are beneficial for both grandparents and grandchildren. A positive grandparent-grandchild relationship can improve the psychological well-being of older adults and be a source of social support, family history, and identity development. Maintaining meaningful interactions can be, however, a challenging endeavor, especially as life events lead to relocating geographically. Grandparents and grandchildren can have different preferences in terms of communication mediums and different assumptions about the real conversational needs of the other.

**Objective:**

In this study, we will investigate the feasibility and effect of sharing memories of older adults with their grandchildren in social media. This intervention focuses on bringing snippets of the lives of the grandparents into the grandchildren’s social media feed and analyzing the potential effect on relational quality, relational investment, and conversational resources from the perspective of the grandchildren.

**Methods:**

A randomized controlled trial will be used to measure the effectiveness of sharing family memories through social media on intergenerational relationships from the perspective of the grandchildren. The study will be implemented in Mongolia among 60 grandparent-grandchild pairs who will be assigned to either a control or intervention group. Pictures and stories will be collected during reminiscence sessions between the researchers and the grandparents before the intervention. During an intervention period of 2 months, grandchildren in the intervention group will receive pictures and stories of their grandparents on their social media account. Pre- and postintervention questionnaires will measure relationship quality, relationship investment, and conversational resources and will be used to assess the effectiveness of the intervention.

**Results:**

We conducted a pretest pilot from January to April 2018 among 6 pairs of participants (6 grandparents and 6 grandchildren). The validation of the protocol was focused on the process, instruments, and technological setup. We continued the study after the validation, and 59 pairs of participants (59 grandparents and 59 grandchildren) have been recruited. The data collection was completed in November 2019.

**Conclusions:**

The results of this study will contribute to strategies to stimulate social interactions in intergenerational pairs. A validation of the study process is also presented to provide further operational recommendations. The lessons learned during the validation of the protocol are discussed with recommendations and implications for the recruitment, reminiscence sessions, technological setup, and administration of instruments.

**International Registered Report Identifier (IRRID):**

DERR1-10.2196/16315

## Introduction

### Importance of Intergenerational Interactions

Vast literature in familial intergenerational interactions is devoted to the study of the grandparent-grandchild relationship. Older adults not only benefit with joy, love, closeness, and company from grandchildren [1], but the expression of affection toward grandchildren is positively associated with psychological well-being (eg, reduction in loneliness and stress and increased general mental well-being) [2]. For younger adults, interactions with grandparents account for the majority of intergenerational interactions [3]. Grandchildren benefit from these relationships affectively, cognitively, and materially [1]. Grandparents are a source of social support, family history and identity, and development [4,5]. However, maintaining and developing intergenerational interactions is a challenging endeavor and there are barriers to interactions.

### Barriers

#### Geographic Separation

Geographic distance is one of the turning points in intergenerational interactions and particularly in the grandparent-grandchild relationship [6], as it limits opportunities for face-to-face interactions. However, studies have shown that grandparents can still experience high levels of relational quality using other means of communication (eg, phone or email) if communication is frequent and especially if initiated not only by the grandparents [7,8].

#### Scant Use of Conversational Resources and Skills

Intergenerational conversations can be challenging, as interlocutors have lived through different life periods and might have different assumptions, conversational skills, and needs [9], which may affect the ability to find and engage on appropriate conversation topics for both. On the ability to engage, the perception of properly accommodating the communication to the other person in the conversation is a predictor of communication satisfaction, liking, and emotional closeness [10]. Failing to accommodate and attune the communication to the interlocutor, however, can lead to patronizing speech, painful disclosures, or underutilization of topical resources [9] that affect the satisfaction and willingness to participate in such interactions [11]. As for finding appropriate topics, the lack of conversation topics is reported as a source of anxiety in intergenerational interactions [12].

#### Asymmetry in Relational Investment

Lack of relational investment is yet another challenge in an intergenerational relationship [6]. It particularly affects young adults transitioning to adulthood, a period in which the responsibilities of growing older are associated with changes in levels of interactions and relational closeness [13]. Grandparents, on the other hand, do keep an interest in their grandchildren’s life, but often refrain from contact, asking questions, or addressing some topics, not wanting to be an annoyance and thus relying on the social skills of the young [14]. Social media have opened an opportunity for older adults to learn more about their grandchildren. However, interactions in this medium are most often not reciprocated by older adults [15].

### Objectives

Interventions to promote social interactions have the potential to address challenges. Systematic reviews summarizing years of research show that computer and internet interventions can be effective in reducing loneliness among older adults [16-18]. However, very few interventions have focused on the specific challenges of intergenerational interactions, especially from the side of the young relatives as they navigate periods of complex social changes. Previous studies on technology-mediated intergenerational communication made different assumptions regarding the younger generation’s relationship with their grandparent and their relational needs: (1) some studies assume that the younger relative is already invested in the relationship and actively seeks contact [19], (2) other studies focus on the opposite problem, that of making the older family members aware of the activities of their younger family members [20,21], and (3) a third body of literature aims to bring older adults online and make online interactions with younger adults more reciprocal [22]. This calls for more research into intervention strategies to improve the relational quality in the grandchild-grandparent relationship by focusing on the challenges faced by the younger population.

In this paper, we present a protocol for a study that explores the effect on relational quality of selectively sharing grandparents’ life stories with grandchildren on social media. We focus on the effect that such intervention can have on grandchildren, and particularly young adults, as changing their communication behavior and investment can have great effects on the relationship. We know from previous work that relationships where grandchildren and grandparents are equally likely to initiate conversations are perceived as more satisfying in terms of frequency and quality by grandparents [7]. A previous study also reported that grandparents tend to rate the relationship as very close while their grandchildren as not having a close connection, suggesting different generational perspectives [23]. Thus, in a period of their lives marked by many social changes, approaches to maintaining or increasing frequency and quality social contact with grandparents and the sense of closeness is of paramount importance.

The sharing of family memories in this context is unidirectional: family memories are used as triggers to give grandchildren topics of conversations, stimulate social interactions, and eventually increase their sense of connectedness. All social interactions with grandparents happen outside social media, either by phone or face to face, and, in principle, without the grandparents having to use technology or have a social media presence. This approach is well suited to scenarios where the practice of reminiscence is widely adopted, such as in nursing homes, where family memories can be used as a bridge to connect grandchildren with their (often frail) grandparents [24,25].

The study is inspired by the practice of reminiscence, the process of recollecting past memories—a practice that is common at all ages [26] and often conducted with older adults due to its many functions and benefits. Webster [27] identifies 8 particular functions: death preparation, identity, problem solving, teach and inform, conversation, boredom reduction, bitterness revival, and intimacy maintenance. Thus, reminiscence serves an important social function in facilitating the sharing of personal memories with others, helping to create bonds between people [28], offering an interesting platform for grandchildren to learn about their grandparents and build family history [5], potentially reconnecting with their grandparents.

In this protocol, we focus on studying the effects of sharing grandparents’ pictures and related stories with grandchildren on social media. Thus, the sharing is mediated by technology. In doing so, we address the following main research question:

- RQ1: Does sharing old pictures and related short stories (from grandparents) with young relatives increase their relational quality?

With this research question, we study the impact of the intervention on the intergenerational relational quality from the perspective of the grandchildren, measured as the feeling of closeness with their grandparents. In addition, we look at two main factors contributing to relational quality and observe if these factors increase as a result of the intervention. More precisely, we address the following secondary research question:

- RQ2: Does sharing old pictures (from grandparents) with the young relatives increase their relational investment?

We particularly focus on relational investment as captured by the impact on the grandchildren’s commitment in their relationship with their grandparents, as well as on the frequency of communication.

- RQ3: Does sharing old pictures (from grandparents) with the young relatives provide them with more topics of conversations and relationship chemistry?

We focus on conversational resources as captured by the impact on the openness and predisposition toward each other (relationship chemistry), as well as on the number and diversity of topics of conversation.

The potential impact of the study is to validate an approach to improving relationship quality between grandparents and grandchildren by empowering grandchildren through the sharing of family stories. This approach can complement existing online and colocated reminiscence technology that generally assume active collaboration of family members to keep the older relatives socially active.

## Methods

### Study Design

The study is designed as a randomized controlled trial (RCT) where both the intervention and control group consist of grandparent-grandchildren pairs. In the intervention group, we collect a set of pictures and short stories about the grandparents and share them with the grandchildren’s social network account, one at a time, at regular intervals across a period of 2 months. In the control group, there is no sharing or collecting pictures as the goal is to account for alternative effects.

**Figure 1:**
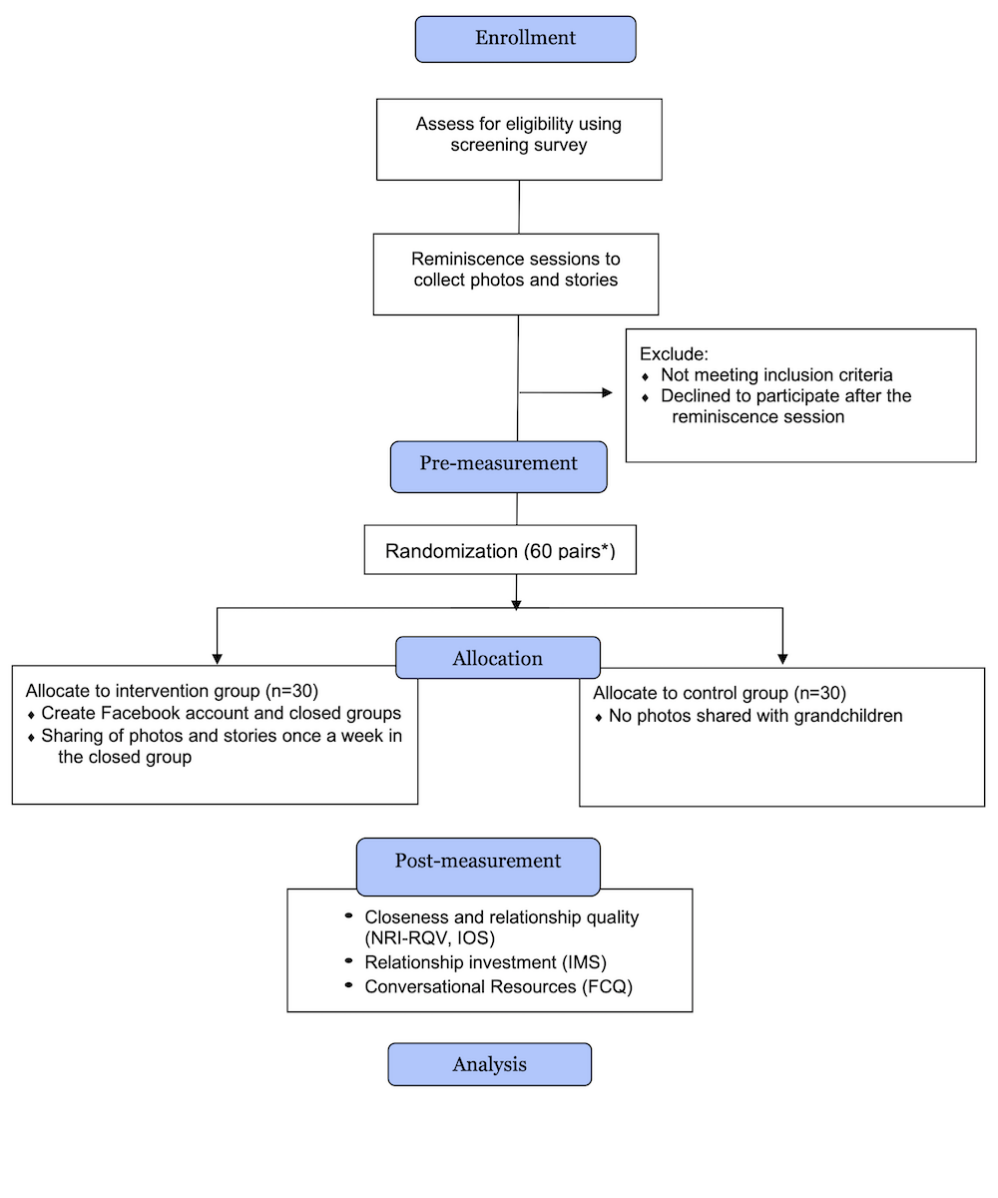
Study Flow. *: 60 grandchildren will be paired with their grandpaerents; NRI-RQV: Network of Relationships Inventory–Relationship Quality Version; IOS: Inclusion of Other in the Self scale; IMS: Investment Model Scale; FCQ: Friendship Chemistry Questionnaire.

Figure 1 shows the diagram of the study flow. Restricted randomization will be performed by stratifying gender, initial closeness level of the grandchildren, and age group of the grandparents. This paper reports on a version of the protocol accepted by the Research Ethics Committee at Mongolian National University of Medical Sciences in December 2017 (No. 2017/3-05).

### Participants

The aim is to recruit 60 grandparent-grandchild pairs, which will be split evenly into intervention and control conditions. The sample size is limited by the specific focus of the intervention and the availability of participants complying with the criteria, but it represents a valid sample for the statistical analyses. A convenience sample will be used in this study to recruit participants. We consider eligible pairs of grandchildren and grandparents meeting the following inclusion criteria.

Grandchildren should be willing to participate in the study and meet the following requirements:

- Aged 18 to 30 years: we recruit young adults because this is a transition period to adulthood associated with changes in the level of interactions and relational closeness [13]
- Be regular users of Facebook: we use Facebook because this is the most common social media among Mongolian young adults [29], and we define regular users as those who check their accounts every 2 to 3 days or more often
- Have infrequent meetings with their grandparent: the proposed intervention is aimed at improving relationships between grandparents and grandchildren who do not have much opportunity to meet and talk. Therefore, we recruit grandchildren who live separately from their grandparents (not in the same apartment or building) and do not meet with their grandparents more than once a week on average

In addition, the grandchildren should have one grandparent willing to participate in the study who meets the following requirements:

- Retired and aged over 60 years: according to Mongolian law, the retirement age is 55 years for women and 60 years for men
- Be able to independently communicate with a researcher: the selected grandparent will be requested to share their life story and give permission to researchers to post photos on Facebook. Therefore, we recruit grandparents who are competent to participate in the study
- Not have any account on social media: the proposed intervention aims at sharing pictures and related stories with the grandchildren in a controlled environment, and thus assumes there is no other form of sharing in the grandparent-grandchild pairs occurring on social networks. Therefore, including grandparents who use social media will not match the study objective
- Have at least 15 available pictures about their life: during the intervention period, we will share Facebook posts 8 times (once a week). Each Facebook post will consist of at least 1 or 2 photos that illustrate a story. In addition, the stories will be about the different life span of the grandparent; therefore, we need 15 photos to illustrate different life stories of the grandparent

### Recruitment

Grandchildren will be recruited from the public universities in Ulaanbaatar, Mongolia, using online as well as paper-based recruitment surveys. As a first step, we will reach students by introducing the study and its goals at the end of their lectures. Those interested in participating in the study will then stay in the classroom to complete the recruitment survey and sign the consent form. The survey will include filtering questions, reflecting the inclusion criteria for grandchildren, and will ask participants to provide an email or phone number for a follow-up. Students who met our inclusion criteria and who have stated their willingness to invite their grandparents to join in our study will be contacted by the researchers to confirm the interest of their grandparents and organize a phone interview. The list of potential candidates will be managed electronically, with access provided only to the researchers in charge of the recruitment.

As a second step, the researchers will contact the grandparents for a phone interview to confirm their eligibility. At the end of the phone interview, a follow-up visit will be scheduled with the grandparents who met the inclusion criteria. We will recruit study participants and make visits until we have 60 pairs that meet the inclusion criteria and agree to participate.

### Processes and Intervention

#### Home Visit to Collect Photos and Stories

Researchers will make a home visit according to the scheduled appointments with the selected grandparents. At the beginning of the meeting, researchers will introduce themselves, explain the purpose of the study, and request that the grandparent read and sign the consent form. Researchers will then ask grandparents to bring pictures that illustrate their life history that they would be willing to share with their grandchildren. In order to ensure diversity in the pool of pictures to share, researchers will propose selecting pictures with the following characteristics: (1) relevant life events such as birth, marriages, work, etc; (2) pictures the grandparents like, find funny or interesting, or that they feel proud of; (3) pictures that include children as well as grandchildren; and (4) pictures that are very old and some more recent. During the visit, researchers will start a conversation regarding the photos by asking the story behind each picture. The researchers will digitize the picture (eg, taking a picture with a mobile phone) and take note of the stories told during the reminiscence session.

#### Premeasurement

Researchers will schedule a meeting with the participating grandchildren to complete the premeasurement questionnaire, which includes all instruments described in the Outcome Measures section.

#### Intervention

The intervention will be implemented over a period of 2 months among the participants of the intervention group. The sharing of pictures and stories will be done using Facebook closed groups. A closed group will be created for each grandchild with whom posts will be shared privately by one of the researchers using an account created for the sole purpose of the study. Grandchildren will be invited to their specific closed group before the start of the intervention with a welcome post explaining in detail the procedure of the picture sharing. A privacy feature of this setup is that sharing outside closed groups is not enabled, which means that grandchildren will not be able to share the original posts outside the closed group.

During the intervention, grandchildren will receive one post per week. The post will be composed by the research team using the pictures and stories collected during the reminiscence session. Each post will include a picture and the related story written as told by the grandparent to the researcher. It is important to note that the older adults will not be directly involved in the intervention process, as this intervention does not enable direct communication between grandparents and grandchildren (ie, grandchildren will not be able to interact with their grandparents through Facebook). Any potential interaction in the grandparent-grandchild pairs will occur through other mediums. Participants in the control condition will only participate in the pre- and postmeasurement sessions. All participants are informed verbally and in the informed consent of their rights, which includes leaving the study at any moment.

#### Postmeasurement

After 2 months of intervention, the grandchildren in both intervention and control groups will be invited for an individual meeting to perform postmeasurement. The same questionnaires provided in the premeasurement session will be used. In addition, the participating grandchildren will be debriefed on their experience during the intervention, and on if and how it affected their relationship with their grandparent.

### Outcome Measures

#### Primary Outcomes

The Inclusion of Other in the Self (IOS) scale and the Network of Relationships Inventory–Relationship Quality Version (NRI-RQV) will be used to measure the relationship of grandparent and grandchild.

IOS is a pictorial tool developed to measure the degree of interpersonal connectedness. In this study, a modified version of the IOS by Gächter et al [30] will be used. This instrument was chosen because it is validated and more comprehensible than the original measure. The NRI-RQV is a combination of the Network of Relationships Inventory and a family relationship measure developed by Buhrmester and Furman [31]. This 30-item survey has 10 scales with 3 items per scale. In this study, the 5 positive features—companionship, intimate disclosure, satisfaction, emotional support, and approval—of the NRI-RQV will be used as a measure of closeness between grandchildren and grandparents. The original questions were adapted by replacing the pronouns with grandparent to fit the grandparent-grandchild relationship.

#### Secondary Outcomes

We will rely on the Investment Model Scale (IMS) [32] and self-reported communication frequency to measure relationship investment. The IMS is an instrument developed to measure commitment level and 3 bases of dependence, which are satisfaction level, quality of alternatives, and investment size. The IMS includes a specific subscale on relationship investment that will be used to measure the grandchild’s contribution in this relationship. The original questions were adapted to the grandparent-grandchild relationship by replacing the word partner with grandparent. Relationship investment scales are scored by computing the scores with higher scores representing higher commitment. Communication frequency between the grandparent and grandchildren will be measured with a custom question with response values going from a few times a week to never.

We rely on the Friendship Chemistry Questionnaire (FCQ) [33] and a custom-made set of questions to measure conversational resources. The FCQ was developed to explore friendship formation factors, comprising 5 subscales: reciprocal candor, mutual interest, personableness, similarity, and physical attraction [33]. In this study, reciprocal candor and similarity scales of the FCQ will be used to estimate available conversational resources, as they reflect on the openness and predisposition toward each other. The original questions were adapted from friendship to the grandparent-grandchild relationship by replacing the pronoun. The FCQ is scored by averaging the items with high scores representing the higher levels of each scale. Two separate questions were redacted and added by researchers to assess general conversational resources and identify common conversational topics with grandparents.

As part of the debriefing interview with participants, researchers will inquire about how the intervention affected their relationship with their grandparent, including recent positive and negative episodes of interaction with grandparents and what pictures and stories captured their attention and led to more interactions. These insights will be contrasted with the online reactions and comments on the posts.

### Statistical Analysis

We will analyze relational quality and closeness (RQ1) with analysis of variance (ANOVA), with group (control, intervention) as between-subject factor and time (pre- and postmeasurement) as within-subject factor. We will compute the main effect for time and the interaction between time and group. In addition, we will analyze the correlation between connectedness and closeness and the factors contributing to higher increases in relational quality, if any.

To determine a statistically significant difference in the relational investment score (RQ2) between control and intervention groups as a result of the intervention, we will perform an ANOVA with group (control, intervention) as between-subject factor and time (pre- and postmeasurement) as within-subject factor. We will compute the main effect for time and the interaction between time and group. In addition, we will explore the factors contributing to higher increases in relational investment, if any.

To determine a statistically significant difference in the conversational resources score (RQ3) between control and intervention groups as a result of the intervention, we will perform an ANOVA with group (control, intervention) as between-subject factor and time (pre- and postmeasurement) as within-subject factor. We will compute the main effect for time and the interaction between time and group. In addition, we will explore the factors contributing to higher increases in conversational resources, if any. The resulting analysis will include an analysis of any unexpected or adverse effects of the intervention.

## Results

### Pilot

In order to validate the protocol and refine the entire intervention process by collecting actionable recommendations for how to execute the various phases, we conducted a pretest pilot from January to April 2018 among 6 pairs of participants (6 grandparents and 6 grandchildren). The validation of the protocol was focused on the process, instruments, and technological setup. We continued the study after the validation, and 59 pairs of participants (59 grandparents and 59 grandchildren) were recruited. Data collection was completed in November 2019.

### Challenges and Lessons Learned

#### Low Recruitment Rate of Study Participants

We recruited study participants at the Mongolian National University of Medical Sciences. We screened approximately 260 students to recruit participants who met the inclusion criteria. Students were excluded because the inclusion criteria “living separately from their grandparents” and “grandparents living in Ulaanbaatar city” were not met. The low rate of recruitment was related to the fact that the number of students who came from the countryside is much higher than that of students who reside in the city. Based on this experience, we recommend extending the recruitment pool to reach young participants who meet the age criteria but are not necessarily affiliated with any university. In addition, disseminating the online recruitment survey is another possible solution to increase recruitment rate.

#### Scheduling Issues in Home Visits

Although older adults were willing to participate in the study, the appointments for home visit (to collect pictures and stories) were in some cases cancelled or postponed because grandparents were sick, had a doctor’s visit, traveled to the countryside, or were celebrating holidays. Therefore, researchers need to consider potential delays in data collection when developing the time frame of the study.

#### Enjoyable But Lengthy Conversation With Grandparents

Older adults were willing to share photos and stories and enjoyed the sessions. Because older adults were eager to share their stories and photos and the interviewers were attentive to this situation and did not rush the interviews, the duration of the conversations was much longer than the 1 hour initially allocated. We recommend researchers account for this aspect when scheduling reminiscence sessions.

#### Managing Preferences While Ensuring Diversity in the Pictures

When selecting the pictures for the study, older adults expressed preferences on certain pictures to share, which affected our diversity criteria for the pictures and stories. To address this situation, we recommend not imposing the choice on the participants but expanding the number of pictures from which to select.

#### Sharing Pictures on Social Media Raised No Privacy Concerns

Grandparents expressed no privacy concerns in sharing the pictures, stating that they knew the pictures were meant to be shared only with their grandchildren. We recommend being transparent about how the pictures will be shared and treated.

#### Managing the Picture and Story Sharing Proved Feasible on Facebook

The setup consisted of a closed group on Facebook accessible only to the grandchildren. This setting was accepted by the participants, and the sharing was carried out without any difficulties. Pictures were shared every week, and grandchildren were interactive using Facebook reactions such as thumbs up or hearts as feedback on the posts. A few of the participants also expressed their gratitude by commenting on the posts.

#### Running the Proposed Study Process Is Feasible

The grandchildren could easily understand and complete the questionnaires. There were no complaints regarding the questionnaire or the set up of the Facebook groups. In addition, some of the grandchildren said that they enjoyed being part of the study because it reminded them about positive memories of their childhood as well as their grandparents. There were no dropouts.

### Alternative Study Designs

We identified two potential issues that led us to propose alternative study designs. The first issue was balancing the intervention and control group (equipoise). The grandchildren who agree to participate in this study will likely do so to experience the intervention. Thus, assigning them to the control group would not fulfill their decision to be involved in this study. To address this potential issue, we propose a crossover design where control and intervention groups would be swapped after the 2-month intervention period so that the control group becomes the intervention group and vice versa. After the crossover, the same intervention process would be applied for another 2 months, allowing all participants to experience the intervention. Incidentally, the second intervention period would serve as a follow-up for the intervention group. The second issue was low rate of recruitment. In case the researchers are unable to randomize 60 pairs of participants at the same time due to low rate of recruitment, a quasiexperimental design would provide flexibility in choosing the control group, allowing those who agree to participate in the study to experience the intervention (to join the intervention group). The potential selection bias in this alternative should be noted, however. May any of the above changes become necessary, amendments to the current version of the protocol will be applied and communicated to the relevant venue.

## Discussion

### Summary

This protocol presents the design of an RCT to assess effects of sharing old pictures with grandchildren on intergenerational relationships with further recommendations to execute the protocol in future studies.

### Strengths

Maintaining intergenerational relationship over time is beneficial to both young and older adults. However, very few interventions focus on the specific challenges of intergenerational interactions, especially from the young adult side. This study proposes an intervention to maintain and improve the relational quality in the grandparent-grandchild relationship using social media. Social media are widely used among the youth and are therefore a promising resource to discover interesting aspects of the life of their loved ones, which could potentially translate in more conversational resources and motivate an increased commitment in the relationship. Our proposed intervention can be potentially implemented to any society with the technological means, without the need for sophisticated tools.

### Limitations

There are number of possible limitations to this study. First, unsuccessful recruitment of study participants, which led us to reconsider the study design, can be a limiting factor. However, the issue can be attributed to the specific demographical characteristics of Mongolia, as the most sparsely populated country, and other countries may see better chances at successful recruitments. In addition, we proposed an RCT design to identify the impact of social media in the intergenerational relationship instead of a more robust multiple armed RCT design. Although the latter would have been more effective in identifying other factors influencing the intervention, its application would have required a larger population sample, which would have added further challenges to an already complicated recruitment process. Therefore, we opted for collecting qualitative data at the end of the study (debriefing session) to identify additional factors in the intervention, thus mitigating the risks involved in recruiting a larger sample. Furthermore, the study will be carried out only in the city; therefore, people who live in rural areas are not included. Finally, the recruitment will be implemented by reaching out only to the grandchildren; therefore, the future studies will consider involving grandparents in the recruitment in order to reduce the selection bias.

### Conclusion

This paper presented the design of an RCT to assess the effects of sharing old pictures with grandchildren on intergenerational relationships. The intervention leverages the practice of reminiscence and social media sharing to empower grandchildren through the sharing of family stories. We focused on grandchildren, and particularly young adults, as changing their communication behavior and investment can have great effects on the relationship with their grandparents. The proposed intervention can be potentially implemented in any society with the technological means, without the need for sophisticated tools. The results from the pretest pilot highlight some practical recommendations for deploying the protocol.

## Appendix

Multimedia Appendix 1 Project evaluation by the funding agency (European Commission). The project evaluation is based on the activities planned, including user studies.

## Abbreviations

ANOVA: analysis of variance
FCQ: Friendship Chemistry Questionnaire
IMS: Investment Model Scale
IOS: Inclusion of Other in the Self scale
NRI-RQV: Network of Relationships Inventory–Relationship Quality Version
RCT: randomized controlled trial
RQ1: research question 1
RQ2: research question 2
RQ3: research question 3
