# Supplementary material for: Effects of Sharing Old Pictures With Grandchildren on Intergenerational Relationships: Protocol for a Randomized Controlled Trial
Source: JMIR Res Protoc. 2020 Apr 30;9(4):e16315. doi: 10.2196/16315 (PMC7226052; doi:10.2196/16315)
Supplement: Multimedia Appendix 1 [file resprot_v9i4e16315_app1.pdf]

Ref. Ares(2015)3468797 - 21/08/2015

Proposal Evaluation Form

EUROPEAN COMMISSION

Horizon 2020 - Research and Innovation Framework Programme

Evaluation Summary Report

Call: H2020-MSCA-RISE-2015
Funding scheme: Marie Skłodowska-Curie Research and Innovation Staff Exchange (RISE)
Proposal number: 690962
Proposal acronym: DREAM
Duration (months): 36
Proposal title: Social Participation for improving emotional, mental, and physical wellbeing in independently living older adults
Activity: ENG

N.	Proposer name	Country	Total Cost	%	Grant Requested	%
1	UNIVERSITA DEGLI STUDI DI TRENTO	IT	288,000	30.92%	288,000	30.92%
2	POLSKO JAPONSKA WYZSZA SZKOLA TECHNIK KOMPUTEROWYCH PJWSTK	PL	144,000	15.46%	144,000	15.46%
3	Ente Ospedaliero Cantonale	CH	13,500	1.45%	13,500	1.45%
4	UNIVERSITY OF NEW SOUTH WALES	AU	0	0.00%	0	0.00%
5	Universidad Catolica Nuestra Señora de la Asuncion	PY	121,500	13.04%	121,500	13.04%
6	UNIVERSITY OF THE PHILIPPINES MANILA	PH	121,500	13.04%	121,500	13.04%
7	University of Costa Rica	CR	121,500	13.04%	121,500	13.04%
8	ASSOCIAZIONE TRENTO RISE	IT	0	0.00%	0	0.00%
9	Mongolian National University of Medical Sciences	MN	121,500	13.04%	121,500	13.04%
	Total:		931,500		931,500	

Abstract:
The combination of longer life expectancy, evolving socio-economical norms and conditions, and new technologies are dramatically changing life after retirement, and not always for the better. In more and more countries, pensioners find themselves with many years in front of them, some of them likely characterized by reduced physical and cognitive abilities. For older adults, this span of time out of the workforce was traditionally devoted to the role (and source of great joy) of caring for grandchildren. However, this role is fading out because of increased mobility of children, which often live far away for work, love, or other reasons, leading to an increase of loneliness and social isolation. This project aims at rethinking long life and understanding the socio-economical context that can make this period of life more exiting and attractive. The specific angle we take is that of enabling older adults of all ages - and specifically including adults who cannot leave their home or that have reduced cognitive abilities - to learn, grow, interact, and contribute to society through ICT. In other words, we aim at enabling adults to be contributors to societal wellbeing. We generically refer to this group of abilities as life participation abilities, and we focus on these aspects because studies tell us that the ability of interacting and feeling useful and helpful to others is essential to a person's wellbeing, sometimes more so than health. The multi- and inter-disciplinary nature of the project provides the perfect environment for collaboration and knowledge transfer. As such, it requires the expertise and perspective that only the consortium as a whole can provide. The synergies that we build in the project will help not only in the materialization of the solutions starting from the original problems, challenges and requirements, but they will also foster the growth of research and innovation skills of the researchers and institutions involved in the project.

Evaluation Summary Report

Evaluation Result

Total score: 92.60% (Threshold: 70/100.00)

Form information

SCORING

Scores must be in the range 0-5.

Interpretation of the score:

*0– The **proposal fails to address the criterion** or cannot be assessed due to missing or incomplete information.*

*1– **Poor.** The criterion is inadequately addressed, or there are serious inherent weaknesses.*

*2– **Fair.** The proposal broadly addresses the criterion, but there are significant weaknesses.*

*3– **Good.** The proposal addresses the criterion well, but a number of shortcomings are present.*

*4– **Very good.** The proposal addresses the criterion very well, but a small number of shortcomings are present.*

*5– **Excellent.** The proposal successfully addresses all relevant aspects of the criterion.Any shortcomings are minor.*

Criterion 1 - Excellence

Score: **4.50** (Threshold: 0/5.00 , Weight: 50.00%)

Note: The following aspects will be taken into account, to the extent that the proposed work corresponds to the topic description in the work programme. If a proposal is partly out of scope, this must be reflected in the scoring, and explained in the comments.
Quality, innovative aspects and credibility of the research (including inter/multidisciplinary aspects)
Clarity and quality of knowledge sharing among the participants in light of the research and innovation objectives
Quality of the interaction between the participating organisations

Strengths:
- The proposal is credible. The life participation abilities and societal well-being are relevant.
- The proposed research area is well defined and is innovative; the instruments that can make the aged people able to rethink and enjoy long life are well described.
- The interaction activities between the participating organisations are well designed and are consistent with the workplan.
- The knowledge sharing activities are well organized; they aim to equip researchers with knowledge and skills in the proposed research area and enhance their innovation skills. A number of multidisciplinary knowledge sharing actions is planned in the proposal.
Weaknesses :
- There is a lack of quantification in the specific objectives in terms of measurable indicators.

Criterion 2 - Impact

Score: **5.00** (Threshold: 0/5.00 , Weight: 30.00%)

Note: The following aspects will be taken into account:
Enhancing research- and innovation-related human resources, skills and working conditions to realise the potential of individuals and to provide new career perspectives
To develop new and lasting research collaborations, to achieve transfer of knowledge between research institutions and to improve research and innovation potential at the European and global levels
Effectiveness of the proposed measures for communication and results dissemination

Strengths:
-The wide interdisciplinary and inter-sectoral elements of the proposal will help researchers acquire new skills and knowledge.
- The secondments between partners and the expected results of the project will improve research and innovation potential at European and international level and will provide new career perspectives to the researchers.
- The project will reinforce international and inter-sectoral research collaborations; due to existing strong experience in international collaborations and high level of scientific expertise, the impact of setting up new research projects is high.
- Self sustainability of the partnership after the end of the project is convincingly addressed.
- The communication and public engagement strategy are appropriate.
- Intellectual property rights and exploitation of results are well articulated.
-The project will reinforce the lasting research collaboration between EU and TC countries as well as between academic and non-academic sectors.

Criterion 3 - Implementation

Score: **4.40** (Threshold: 0/5.00 , Weight: 20.00%)

Note: The following aspects will be taken into account:
Overall coherence and effectiveness of the work plan, including appropriateness of the allocation of tasks and resources
Appropriateness of the management structures and procedures, including quality management and risk management
Appropriateness of the institutional environment (infrastructure)
Competences, experience and complementarity of the participating organisations and institutional commitment

Strengths:
- Tasks and deliverables are well scheduled.
- The management structure is professionally organised with well established responsibilities.
- Research and managerial competence and experience of the key-staff involved are well described.
-The consortium demonstrates a strong capacity to perform the required tasks.
The institutional environment is appropriate.
Weaknesses:
- No milestones are foreseen for monitoring the work progress.

Operational Capacity

Status: **Operational Capacity: Yes**

Not provided

Proposal content corresponds, wholly or in part, to the topic description against which it is submitted, in the relevant work programme part

Status: **Yes**

Not provided
